# Practical Notes and Formulæ

**Published:** 1878-02-20

**Authors:** 


### PRACTICAL NOTES AND FORMULÆ.

**DISEASES OF THE TEETH, and their influence on Diseases of the Human System.—By O. E. NEWTON, M.D., Cincinnati, O.**

It is true this subject interests, perhaps, more the dentist than the physician; but there are many cases which will be more successfully treated if a full knowledge of the relation of diseased teeth to the surrounding tissue is had. For instance, you will often find a severe neuralgic condition will be the result of some abscess at the root of the tooth, or some exposed nerve in a cavity of a tooth. You may have an ulcerated opening from the bottom of the alveolar cavity under a tooth, allowing a disease of the autrum himoreanum, attended with a deep, severe pain, with swelling of the face, when by removing this tooth and injecting a stimulating fluid through the alveolar opening, a cure will be effected. There will often be such phosphatic deposit around the gum and about the base of the teeth and snags—in the form of tartar—as to afford a sufficient poisonous odor, which, when continuously inhaled, will be a cause of diseased action so as to prevent you being able to cure the case without the mouth be put in a more cleanly condition.

*A Case in Practice.*—Some years ago a country lady became my patient, who remained in the city for treatment for an indolent ulcer upon the front of the upper arm, at about its middle. I laughed at the failure of other physicians to cure it. Among others who tried to cure it, was a distinguished Cincinnati surgeon, who had had a three months' siege at it. I treated the case for two weeks without the least benefit. One day while laughing at some remark I made, I noticed a bad condition of her mouth, filled with decayed snags, covered with tartar, producing an offensive secretion. A dentist cleaned her mouth, and in two weeks more, with the same treatment previously used, the case was discharged, cured.

This case was always a lesson of advice to me to look at the mouth when examing any chronic case of disease.

GODFREY'S CORDIAL.

| | |
|---|---|
| Tinct. opium | 1½ pints. |
| Molasses | 16 " |
| Alcohol | 2 " |
| Water | 26 " |
| Carb. potassium | 2½ ounces |
| Oil sassafras | ½ ounce. |

Dissolve the carbonate of potassium in the water; add the molasses, and heat over the fire until they simmer. Take off the scum which rises, and add the laudinum and sassafras, having previously mixed them well together.

CONDITION POWDER FOR HORSES.

| | |
|---|---|
| Powdered black antimony | 4 ounces |
| Powdered East India ginger | |
| Powdered nitrate of potassinm | |
| Powdered sulphur | |
| Bi-carbonate soda | aa 8 ounces |
| Glauber's salt | 12 ounces |

M. Dose — one tablespoonful to a pound of feed.

The above preparation for horses has come into such general use in the country that it is well for the practitioner to know what it is.

A VERY popular nerve and bone liniment is prepared as follows:

| | |
|---|---|
| Aqua ammonia | 1 ounce |
| Olive oil | 2 ounces |
| Camphorated oil | 1 ounce |
| Oil rosemary | ½ ounce |

NEW PHYSIOLOGICAL PROPERTY OF STRYCHNIA.

It is asserted that strychnia, by increasing the arterial pressure, increases the secretion of the mammary glands in some cases as much as fifteen-fold.

CHILBLAINS.

R.—Collodion ........................ dr. vj.

Tr. ferri chloridi .................... dr. ij.

Ol ricini ........................... dr. v.

Paint with a camel's hair pencil two or three times a day.

HYSTERIA.

Dr. Dabney, of Virginia, advises the following as an excellent combination for hysteria:

R.—Tinc. cinchona bark .................. oz. ij.

Tinc. nux vomica

Dilute phosphoric acid ............ aa oz. ss.

S. Dose, half a teaspoonful three times a day.

EXCELLENT PODOPHYLLIN PILL.

R.—Podophyllin

Ext. hyosciami .................... aa gr. iij.

Saponis .............................. gr. ivss.

Syrupi simp ............................ gtt. vj.

Div. in pil. No. xii.

Dose, one to two pills.—*West. Lancet.*

BLACK HAIR DYE WITHOUT SILVER.

The following is said to give a good and natural-looking dye, free from the caustic action of silver salts and the poisonous effects of lead compounds. Two preparations are needed:

No. 1.

Citrate of bismuth ..................... 1 ounce.
Rose water ............................. 2 ounces.
Distilled water ......................... 2 ounces.
Alcohol ................................ 5 drachms.
Ammonia, sufficient.

No. 2.

Hydrosulphate of soda ............... 12 drachms.
Distilled water ......................... 4 ounces.

Each solution is to be applied separately.—*Drug. Circular.*

SYR. CITRATE OF IRON AND QUINIA.

The above is an admirable preparation in chronic ague and other low states of the system. It is prepared as follows:

R.—Citrate of iron and quinia, ......... gr. xxxii.
Syrup, warm .............................. oz. iv.
Dissolve by trituration.

Dose, one to three drachms.

CYANIDE OF ZINC.

Dr. W. F. Barr, of Virginia, writes: "In the July number, 1875, of THE SOUTHERN MEDICAL RECORD, p. 437, is published an item on "The Cyanides in Acute Articular Rheumatism, by M. Luton's method: one and one-half grains daily of Cyanide of zinc." In the November number, 1877, p. 305, I notice a selection that Dr. Luton gives the following in "rheumatism and neuralgia," sciatica, etc.:

" R.—Zinc cyanide ........................ grs. iii.

Aq. distillatæ ......................... dr. viss.

Mix —Mulag. acaciæ ...................... dr. iv.

Dose—One Tablespoonful every hour. Shake well before using."

Will it do to give such doses of cyanide of zinc every *hour?* Is there no misprint? If there should be a mistake about the dose, I think that it should be corrected, for there are so many physicians—*too many*—who prescribe by *formulas*—let others do their thinking for them.

[The above formula was given as we found it. We agree with our friend that the dose is too large. A teaspoonful of the mixture would be sufficient, and should not be so frequently given.—ED.]

SACCHARATED CALOMEL.

At the request of a medical friend, we reproduce the formula of saccharated calomel:

R.—Calomel ............................. grs. x.
Loaf sugar ............................. grs. xl.
Ttiturate thoroughly and divide in powders No. xx.

Calomel triturated with sugar is very greatly increased in its purgative action, probably due to the minute division of its molecules. It has been asserted that this increased power of the remedy results from the formation of corrosive sublimate through chemical action. We are disposted to doubt this, yet it may be well not to use the article too freely. Certain it is, that very minute quantities of calomel in this shape acts with promptness and efficiency.

ARSENICAL PASTE.

Amongst the various caustic preparations used for the destruction of malignant tumors, arsenic is perhaps the most relied upon.

A variety of pastes have been devised for this purpose, with the use of which Cancer Doctors have obtained more or less celebrity. Arsenic possesses the peculiar property of not destroying the healthy tissues.

Cosme's paste, as modified by Hebra, is regarded as the best form of preparing it.

R.—Acidi Arseniosi.......................grs. xx.
Hydrarg. sulphuret, rub.................dr. j.
Ungt. simplicis..............................ozj.
M. Ft. Ungt.

This is used by spreading upon a cloth and applying to the part every day, occasionally suspending and detaching the rochar by poulticing. It is a very painful process.

TUPENTINE IN TYPHOID FEVER.

The following formula for the administration of turpentine has acquired great celebrity in the Dublin hospital:

R.—Terebinthinæ olei

Liquoris potassæ...................... aa dr. ij.

Mucilag accaciæ......................dr. iv.

Syrupi papaveris albi,

Syrupi floris aurantii.................. aa oz. i.

Aquæ camph. q. s. ad............ oz. viij.

Fiat misiura—Dose, a tablespoonful every four hours, shaking the bottle.

DALBY'S CARMINATIVE.

R.—Carb· magnesia.................. 40 grains.

Oil of peppermint................ 1 drop.

Oil of anise.............................. 3 drops.

Tinc. of castor.......................... 30 drops.

Tinc assafœtida................ 15 drops.

Tinc. Opium.................... 5 drops.

Essence pennyroyal.............. 15 drops.

Com. tinc. cardamon.............. 30 drops.

Peppermint water.................... 2 ounces.

Rub the oils with the magnesia, then add the peppermint water and the rest of the ingredients. Shake and give 20 to 60 drops to relieve coloic and flatulence in children.

ELIX. OF VALEREAN.

R.—Aromatic elixir.......................... 15¼ oz.

Fluid ext. valerian.......................... ¾ oz.

Mix. Dose, one tc two drachms.

YELLOW WASH.

R.—Corrosive sublimate ............ 18 grains.
Lime water............................ 10 ounces.
Mix.

DIABETES.

A writer in *Eclectic Medical Journal*, recommends Lycopus Virginicus, (or bugleweed) as a remedy of unusual efficacy in diabetes:

R.—Lycopus Virginicus, bugleweed. Make an infusion of the fresh plant about one ounce to a pint of hot water. Dose, a tablespoonful four or five times daily.

MENORRHAGIA.

Dr. Racebonski's remedy of Paris is the following:

R—Iron by hydrogen......................dr. j.
Extract of nux vomica..............grs xii.
Mucilage gum arabic.................. q s
Divide into sixty pills.

From two to four morning and evening, for chlorotic young girls whose menstruation is too profuse.—*Naphey.*

CHURCHILL'S TINC. IODINE.

R—Tinc. Iodinii
Acid Carbol. liq ................ aa 1 part
Hydrate Chloral................... 2 parts

This preparation is caustic, and has been used in ulcerations of the os uteri.

A SUBSTITUTE FOR LITMUS PAPER.

As a substitute for ordinary test paper, Dr. E. Luck draws attention to a new substance, phenol-phtalein, which may be easily prepared by heating phenol with phtalie anhydride and concentrated sulphuric acid. This body is entirely colorless in neutral or acid solutions, but exhibits an intense purple color in the presence of the least excess of alkali. The change of color is instantaneous, and its depth intense, so that even mere traces of the indicator and of an alkali become recognizable.—*Med. Reporter.*

